# In-hospital unexpected cerebral herniation—New neurosurgical quality control standards: A case report

**DOI:** 10.1097/MD.0000000000044855

**Published:** 2025-10-03

**Authors:** Liyang Hang, Xingming Zhong, Yong Cai

**Affiliations:** a Department of Neurosurgery, the First People’s Hospital of Huzhou, Zhejiang, China.

**Keywords:** cerebral herniation, inpatient management, quality control

## Abstract

**Rationale:**

In-hospital unexpected cerebral herniation (IHUCH) refers to abrupt, unanticipated cerebral herniation in patients who were previously considered stable. Its sudden onset may lead to delayed recognition and poor outcomes.

**Patient concerns:**

A 50-year-old man was transferred to our institution after a motor vehicle collision. The patient’s Glasgow Coma Scale score remained between 13 and 14 upon admission. At the sixth day, without warning, he acutely lost consciousness, dropping to Glasgow Coma Scale 6, with left pupillary dilatation and loss of light reflex.

**Diagnoses:**

Initial computed tomography revealed: left frontotemporal traumatic subdural hemorrhage, right-sided epidural hemorrhage, traumatic subarachnoid hemorrhage, right temporal bone fracture, left traumatic intraparenchymal hematoma, pneumocephalus, and basal skull fracture. Without surgical indications, day 6 emergency computed tomography demonstrated the compression of the left lateral ventricle with rightward midline shift, effacement of the suprasellar cistern and perimesencephalic cisterns, and marked cerebral edema.

**Interventions:**

Urgent left decompressive craniectomy with evacuation of subdural hematoma was performed. Postoperatively, the patient’s intracranial hypertension remained refractory. As conservative measures failed, a contralateral decompressive craniectomy was performed the next day, opposite the initial surgical site.

**Outcomes:**

Despite prompt surgical decompression, the patient remained in a persistent comatose state. After 5 months of comprehensive neuro-rehabilitation without neurological improvement, the family elected to withdraw life-sustaining treatment and the patient was discharged home.

**Lessons:**

This case illustrates how IHUCH can occur even under close neuro-monitoring. Key risk factors include postoperative hematoma recurrence, bilateral frontal lobe contusions, delayed traumatic intracranial hematoma, paradoxical cerebral herniation, chronic subdural hygroma, and tumor. Integrating IHUCH into neurosurgical quality control standards, augmented by continuous multimodal intracranial pressure monitoring, may enhance early detection and improve outcomes.

## 1. Introduction

Cerebral herniation constitutes a prevalent and acute critical condition encountered in the clinical realm of neurosurgery. Its mechanism primarily arises from substantial alterations in intracranial pressure (ICP) coupled with pressure disequilibrium among distinct brain regions, leading to the displacement of cerebral parenchyma. Subsequently, affected cerebral parenchyma protrudes through physiological apertures or fissures into regions exhibiting lesser pressure, thereby eliciting an array of grave neurological dysfunctions.^[1]^ Typical manifestations of cerebral herniation include the progressive loss of consciousness, pupillary changes, limb paralysis, and unstable vital signs. If cerebral herniation is not promptly recognized and treated, it can rapidly progress to respiratory and circulatory failure, thereby posing a significant threat to the patient’s life.^[2]^ Therefore, neurosurgeons must possess a thorough understanding of the pathophysiological mechanisms, clinical manifestations, and imaging characteristics of cerebral herniation. This knowledge enables timely diagnosis and implementation of comprehensive therapeutic interventions in clinical practice, thereby reducing mortality and morbidity rates while optimizing patient prognosis. However, it should be noted that the onset of cerebral herniation may demonstrate a degree of clinical unpredictability, with certain patients progressing rapidly to this critical condition even in the absence of overt warning signs.^[3]^ Given the sudden onset and clinical unpredictability of such cerebral herniation events in clinical practice, we cautiously propose a novel conceptual framework termed “in-hospital unexpected cerebral herniation (IHUCH)” to describe cases where hospitalized patients abruptly develop cerebral herniation without prior anticipation by health-care providers. Currently, only limited clinical studies and discussions address this specific patient population. To address this knowledge gap, we advocate for the inclusion of IHUCH in neurosurgical quality control standards to enhance academic attention and reduce its incidence. This report presents a representative case to illustrate the proposed conceptual framework.

## 2. Case

Written informed consent was obtained from the patient’s next of kin for publication of this case report and any accompanying images.

**Figure 1.**
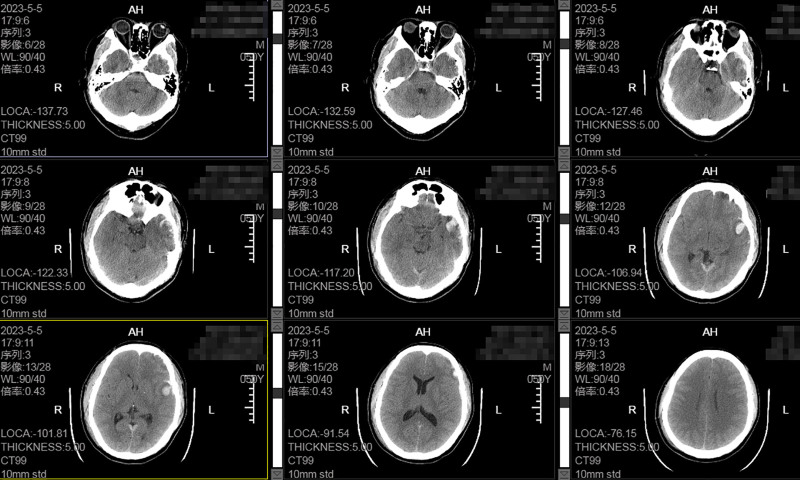
The initial CT scan upon admission revealed a traumatic subdural hematoma in the left frontotemporal region. The intracranial condition was stable, with no significant mass effect or midline shift, and there were no surgical indications. Based on these findings, conservative treatment was initiated. CT = computed tomography.

A 50-year-old male patient was transferred to our institution following traumatic injury sustained in a motor vehicle accident. Upon admission, the patient exhibited impaired consciousness and incoherent responses, with active hemorrhage from the right external auditory canal, nausea, and vomiting. His Glasgow Coma Scale (GCS) score was between 13 and 14. An emergency computed tomography (CT) scan was performed, revealing the following findings: a right temporal bone fracture accompanied by a small ipsilateral epidural hematoma. Concurrently, subdural hematoma was observed in the left frontotemporal region, with an adjacent small intraparenchymal hematoma within the left frontal lobe. Additional findings included subarachnoid hemorrhage and minimal intracranial pneumocephalus (Fig. 1). Based on these findings, the patient was initially diagnosed with: left frontotemporal traumatic subdural hemorrhage, right-sided epidural hemorrhage, traumatic subarachnoid hemorrhage, right temporal bone fracture, left traumatic intraparenchymal hematoma, pneumocephalus, and basal skull fracture.

Given the absence of surgical indications, the patient was managed conservatively with intensive neuro-monitoring. Serial CT scans demonstrated persistent compression of the left lateral ventricle, midline shift to the right, and bilateral frontotemporal soft tissue swelling. The GCS score remained stable at 13 throughout the initial 5-day observation period.

**Figure 2.**
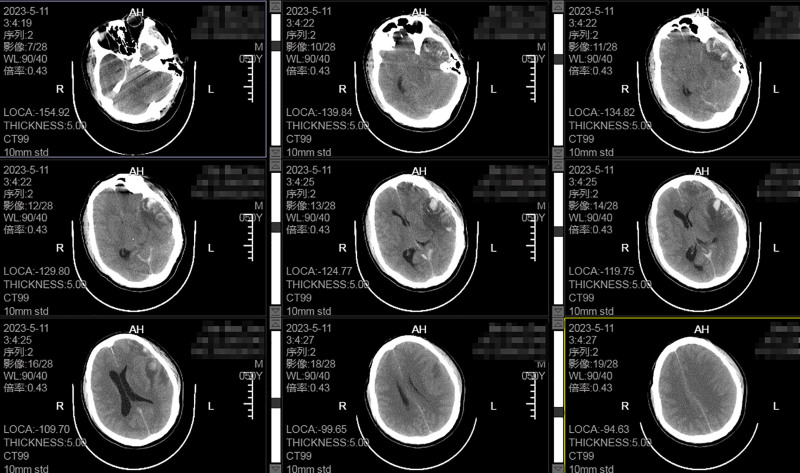
The preoperative CT scan demonstrated compression of the left lateral ventricle, rightward midline shift, obliteration of the suprasellar cistern and perimesencephalic cisterns, and significant cerebral edema, indicating the necessity for surgical intervention. CT = computed tomography.

**Figure 3.**
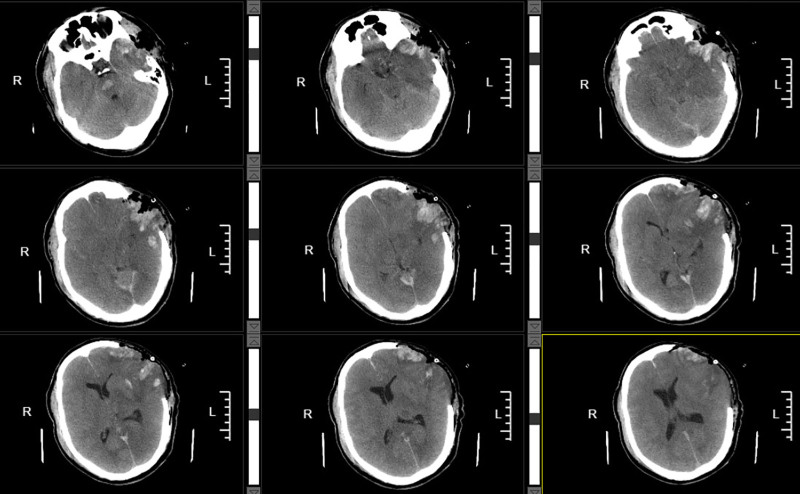
CT scan demonstrated restoration of midline structures, preservation of the ambient cisterns, and evidence of hemorrhage within the decompressed brainstem. CT = computed tomography.

On the morning of hospital day 6, follow-up cranial CT revealed stable intracranial pathology. The patient’s GCS score remained at 13. As of this point, the patient’s CT Marshall classification has remained at Grade 2, with bilateral pupillary reactivity consistently maintained. The GCS-P score has also been sustained around 13. However, acute neurological deterioration occurred at 2:40 am, manifesting as sudden loss of consciousness (GCS 6), anisocoria (left pupil 5 mm vs right 4 mm, bilateral light reflex absent), and inability to respond to motor commands. Intravenous mannitol was administered emergently to reduce ICP, and all necessary preparations were expedited for immediate transfer to the CT suite. A pre CT scan was successfully acquired at 03:04. Emergency CT demonstrated the compression of the left lateral ventricle with rightward midline shift, effacement of the suprasellar cistern and perimesencephalic cisterns, and marked cerebral edema (Fig. 2).The preoperative consultation was completed at 03:11. An emergent left-sided craniotomy for hematoma evacuation and decompressive craniectomy was performed under general anesthesia, followed by admission to the intensive care unit. After the patient’s condition stabilized, a repeat CT scan revealed complete evacuation of the postoperative hematoma, restoration of midline structures, and reappearance of the ambient cisterns; however, decompressive hemorrhage within the brainstem was noted (Fig. 3).

**Figure 4.**
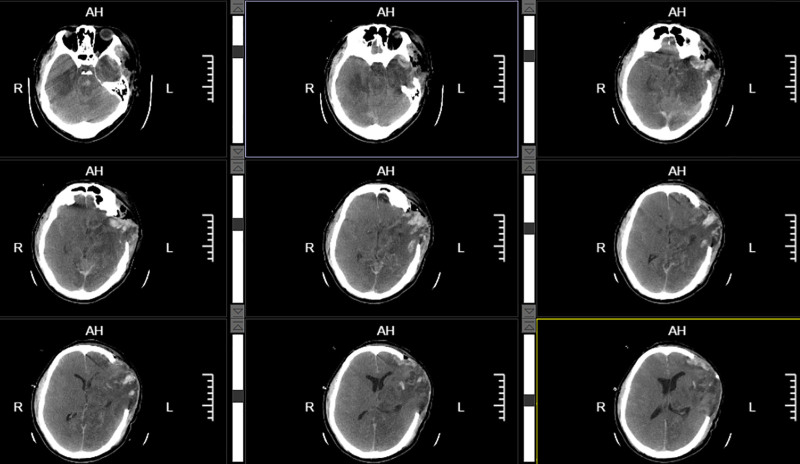
CT imaging revealed obliteration of the ambient cisterns, diffused cerebral edema, compression of the brainstem, and marked signs of intracranial hypertension. CT = computed tomography.

**Figure 5.**
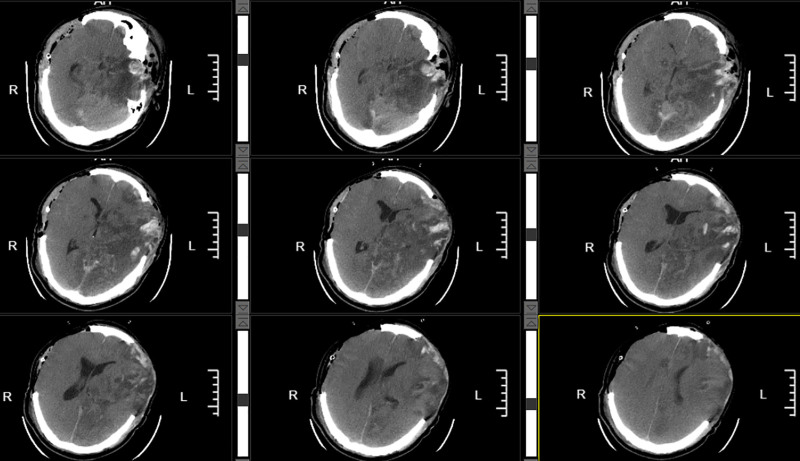
CT imaging demonstrated essentially preserved midline structures, faintly discernible ambient cisterns, and evidence of alleviated intracranial hypertension. CT = computed tomography.

Despite continuous intracranial-pressure-lowering therapy, including mannitol administration and other modalities, initiated after the first operation, the response was suboptimal. A follow-up CT scan obtained at 10:39 on the second postoperative day revealed the obliteration of the ambient cisterns, diffuse cerebral edema, and brainstem compression (Fig. 4). On the afternoon of postoperative day 2, contralateral pupillary dilation was observed. The patient remained in a comatose state, with the left pupil measuring 4.5 mm and the right 3.5 mm. In light of the critical nature of this decision, the indication for reoperation was deliberated in a multidisciplinary discussion, after which informed consent was obtained from the patient’s legal surrogates. An emergency right-sided decompressive craniectomy was subsequently performed at 14:15 on the same day. Once the patient’s condition had stabilized, a follow-up CT scan was obtained. Imaging revealed restoration of midline structures with only faint visualization of the ambient cisterns, indicating effective alleviation of intracranial hypertension (Fig. 5).

Postoperatively, the patient remained in a persistent comatose state throughout the 5-month therapeutic course. Following comprehensive evaluation demonstrating no significant clinical improvement, the family elected to withdraw life-sustaining interventions, after which the patient was discharged from hospital care.

## 3. Discussion

Herniation of the brain is a severe and relatively common condition in the field of neurosurgery, often associated with changes in ICP. In a retrospective study, researchers observed that the incidence of cerebral herniation in patients with moderate to severe intracerebral hemorrhage could be as high as 46%.^[2]^ This data highlights the need for heightened clinical vigilance regarding cerebral herniation. However, during hospitalization, some patients may experience cerebral herniation that is not entirely within the anticipation of medical staff. We define such cases as IHUCH. Once this phenomenon occurs, it significantly impacts patient prognosis. Given its severity, the medical community should give this issue greater attention.

Since the concept of IHUCH has not yet been incorporated into any quality control standards in neurosurgery, we propose that it be included in the quality control criteria for neurosurgery to enhance the medical community’s focus on this phenomenon. The etiology of IHUCH is not isolated; rather, it is multifactorial, with numerous factors potentially contributing to its occurrence. The following are several common factors that may induce IHUCH.

### 3.1. Postoperative hematoma recurrence

Postoperative hematoma recurrence refers to the reemergence of new hemorrhage or expansion of the initial hematoma in the surgically managed area during the recovery phase, often leading to elevated ICP and aggravated neurological deficits. In specific patient populations, the incidence may reach 10% to 40%, with direct correlations observed between hematoma recurrence and unfavorable clinical outcomes.^[4]^

The occurrence of recurrent hemorrhage may be associated with premature surgical intervention. A study demonstrated that patients undergoing craniotomy within 4 hours post-onset exhibited significantly higher mortality rates compared with those receiving surgical treatment within 12 hours.^[5]^ Suboptimal postoperative blood pressure control constitutes another critical factor contributing to hematoma recurrence, potentially attributable to chronic hypertension-induced cerebrovascular pathology and necrotic changes. This pathological alteration results in diminished vascular wall elasticity, facilitating persistent blood extravasation through compromised vasculature. Notably, rigorous blood pressure management has been correlated with a lower incidence of hematoma recurrence.^[6]^ The “spot sign” observed in CT angiography has been identified as a valuable predictor for hematoma expansion.^[7]^

### 3.2. Bilateral frontal lobe contusions

Bilateral frontal lobe contusions, frequently encountered following motor vehicle accidents and falls, may result from direct traumatic impact to the frontal region or contrecoup injury mechanisms originating from occipital impacts.^[8]^ The resultant hematomas or edema formation in bilateral frontal regions can induce localized elevated ICP.^[8]^ This pathophysiological disturbance propagates posteriorly, precipitating central transtentorial herniation with consequent lethal midbrain compression.^[9]^ Clinically, such mechanisms account for sudden neurological decompensation in previously stable patients, occasionally progressing to mortality within abbreviated timeframes.^[10]^ These critical scenarios pose substantial challenges in clinical management, necessitating heightened clinical vigilance to mitigate adverse outcomes. The implementation of ICP monitoring^[8]^ combined with blood flow measurements^[10]^ constitutes an effective preventive strategy against the development of central herniation.

### 3.3. Delayed traumatic intracranial hematoma

Delayed traumatic intracranial hematoma (DITCH) is defined as intracranial hemorrhage undetected during initial neuroimaging (typically CT) following head trauma but subsequently identified upon repeat examinations conducted hours to days later. As a prevalent complication of craniocerebral injury, scholarly investigations have documented DITCH incidence rates reaching 7% among head trauma populations.^[11]^

Severe DITCH manifestations exert a significant mass effect on adjacent cerebral parenchyma, with this compressive pathophysiology constituting a critical mechanism underlying cerebral herniation development, hematoma expansion induces progressive intracranial hypertension, creating pressure gradients that drive structural displacement of brain tissue. Robert Ziechmann et al categorized DTICH into 3 principal types and 1 subtype, accompanied by etiopathogenetic explanations for each category.^[12]^

### 3.4. Paradoxical cerebral herniation

Paradoxical cerebral herniation is characterized by the displacement of brain tissue into the cranial defect region following decompressive craniectomy, resulting from atmospheric pressure exceeding ICP. This displacement ultimately precipitates cerebral herniation. There is a distinct correlation between paradoxical cerebral herniation and decompressive craniectomy. In patients with skull defects, procedures involving cerebrospinal fluid drainage, such as lumbar puncture,^[13]^ ventriculoperitoneal shunting,^[13]^ and lumbar cistern drainage,^[14]^ may further induce the occurrence of paradoxical cerebral herniation. Moreover, the average daily volume of cerebrospinal fluid drainage is likely an important risk factor for the development of paradoxical cerebral herniation.^[14]^

Paradoxical cerebral herniation can occur months or even longer after decompressive craniectomy. The early symptoms of this condition are nonspecific and may manifest as sudden motor weakness, reduced speech, and memory impairment, which can easily lead to misdiagnosis or missed diagnosis. This underscores the need for clinicians to be highly vigilant about this potential complication. Early cranioplasty can effectively prevent the occurrence of paradoxical cerebral herniation.^[15]^ Once paradoxical cerebral herniation occurs, timely intravenous fluid resuscitation and positioning the patient in the Trendelenburg position can effectively improve symptoms and provide an opportunity for further treatment.^[15]^

### 3.5. Chronic subdural hygroma

Chronic subdural hygroma is characterized by abnormal fluid accumulation within the subdural space between the dura mater and arachnoid membrane. While conventionally attributed to traumatic etiology, emerging evidence suggests a more complex pathophysiological mechanism.^[16]^ In chronic presentations, neomembrane formation within the subdural space may restrict the flow of subdural effusion. Paradoxically, this membrane can serve as a potential herniation conduit through which cerebral tissue may protrude into the subdural compartment, precipitating catastrophic neurological sequelae.

This phenomenon demonstrates higher prevalence in pediatric populations, with postulated associations to enhanced cerebral plasticity secondary to incomplete myelination. In adult cohorts, postprocedural localized cerebral edema following surgical evacuation of hematomas has been implicated as a predisposing factor for herniation events.^[17]^

### 3.6. Tumor

Intracranial tumors are a common condition in neurosurgery. The growth of intracranial tumors can lead to increased ICP, which in turn compresses normal brain tissue and may precipitate cerebral herniation. It is noteworthy that patients with relatively stable intracranial tumors may experience intratumoral hemorrhage without obvious precipitating factors^[18]^ or following trauma.^[19]^ These newly formed intracranial hematomas can rapidly cause an imbalance in ICP, leading to cerebral herniation.

Having explored the underlying mechanisms and contributing factors associated with IHUCH, it is essential to transition our focus toward the development and implementation of effective preventive measures.

Prevention of in-hospital cerebral herniation necessitates the implementation of multimodal ICP monitoring protocols. Conventional clinical paradigms emphasize serial CT surveillance at predetermined intervals during acute phases, coupled with meticulous pupillary change observation, as primary measures for mitigating most in-hospital herniation events. However, these strategies demonstrate inherent limitations.

In specific patient cohorts, cerebral herniation exhibits temporal unpredictability, maintaining risk potential throughout hospitalization. It is imperative to emphasize that the frequency of CT reassessment is relatively high in the early stages of hospitalization, and it gradually decreases over time. This temporal disparity in neuroimaging surveillance may predispose to cerebral herniation oversight during the latter period, thereby amplifying diagnostic omission risks.

Although the patient treated in our hospital did not meet the criteria for admission to the intensive care unit (ICU) and appeared to have a favorable prognosis, a fatal disease outcome still occurred. This highlights that the clinical course of patients with traumatic brain injury can be highly unpredictable and may deteriorate rapidly. Even patients with mild to moderate severity are at risk of experiencing significant clinical deterioration within a short period. For patients undergoing treatment in specialized neurosurgical wards, it is imperative to enhance the frequency of monitoring. Early detection of changes in clinical status and timely intervention may be crucial in improving prognosis. During early herniation stages, pupillary dimensions frequently remain normotypic, whereas fixed pupillary dilation typically manifests post-herniation onset, indicating critical neurological deterioration. Furthermore, pupillary alterations occasionally lack pathological specificity. Ipratropium bromide administration via nebulizer masks may induce pharmacological mydriasis through ocular exposure, mimicking herniation-related pupillary changes.^[20]^ Such iatrogenic artifacts risk precipitating misdiagnosis, potentially triggering unnecessary therapeutic interventions.

Integration of continuous multimodal ICP monitoring with conventional CT/pupillary protocols optimizes herniation prevention efficacy. Invasive ICP monitoring is a well-established technique; however, due to the potential for complications, its application is restricted to specific patients. Currently, non ICP monitoring technologies are rapidly evolving and hold promise for broader clinical applications in the future.^[21]^

Beyond patient-level factors, institutional workflow may influence IHUCH occurrence. In some health-care systems, China being one example, general intensive care units are customarily led by internal-medicine teams that operate separately from the neurosurgical service. Such structural separation can create discrepancies in the intensity of neurological monitoring and in the timeliness of ICP-directed interventions. Recognizing this system-level dimension, we propose that IHUCH be adopted as a joint neurosurgical–ICU quality indicator. The intent is not retrospective censure, but prospective evaluation of whether early, protocolized neurosurgical involvement within the ICU can reduce the incidence of unexpected herniation events and thereby inform evidence-based refinement of neurocritical-care pathways.

## 4. Conclusion

Currently, in all neurosurgical specialty hospitals, key indicators such as infection rates and rates of unplanned reoperations are prioritized as critical benchmarks. However, few hospitals currently incorporate the occurrence of IHUCH into their quality control standards. We anticipate that in the future, a new neurosurgical quality control standard, IHUCH, will be proposed in the quality control work of neurosurgery to reduce its occurrence.

## Author contributions

**Project administration:** Yong Cai.

**Supervision:** Yong Cai.

**Writing – original draft:** Liyang Hang.

**Writing – review & editing:** Xingming Zhong.
